# Gain and Bandwidth Enhancement of 3D-Printed Short Backfire Antennas Using Rim Flaring and Iris Matching

**DOI:** 10.3390/s24082654

**Published:** 2024-04-22

**Authors:** Yewande Mariam Aragbaiye, Dustin Isleifson

**Affiliations:** 1 Department of Electrical & Computer Engineering, University of Manitoba, Winnipeg, MB R3T 5V6, Canada; aragbaiy@myumanitoba.ca; 2 Centre for Earth Observation Science, University of Manitoba, Winnipeg, MB R3T 2N2, Canada

**Keywords:** 3D printing, flare, gain enhancement, iris, short backfire antenna, wideband antenna

## Abstract

In this article, we present new design techniques to improve the gain and impedance bandwidth of short backfire antennas. For the gain enhancement procedure, our approach was to flare the rim of the antenna, which simultaneously led to an increase in the impedance bandwidth of the antenna. Parametric studies were carried out to obtain the optimal flaring angle. The peak realized gain was obtained as 17.2 dBi with an impedance bandwidth of 55% (2.4 dB and 28.6% increase in gain and bandwidth, respectively, compared to the unflared antenna). To further enhance the impedance bandwidth, an inductive iris was added to improve impedance matching at the waveguide aperture. We varied the width of the iris to obtain the optimal width that provided the best gain and impedance bandwidth result of 17.1 dBi and 66% (~40% increase compared to the unflared antenna without iris). To experimentally verify the work, prototypes were fabricated and tested. We found good agreement between simulation and measurement. The results of this study indicate that gain and bandwidth can be enhanced through optimized geometrical modification of the SBF structure. Furthermore, our 3D-printed technique demonstrates a mass reduction compared with conventional metallic structures.

## 1. Introduction

H. W. Ehrenspeck introduced the traditional short backfire antenna (SBF) with circular reflectors for the first time in 1962 at the Air Force Cambridge Research Centre in Bedford, Massachusetts, as an improved version of the backfire antenna. The short backfire antenna was initially published in 1965, and Ehrenspeck was awarded its first patent in 1969 [1,2]. The SBF antenna can produce moderate gain values between 13 dBi to 20 dBi, with sidelobes of at least 10–15 dB and a cross-polarization of under 20 dB. The compact construction, radiation properties, and simplicity of the feed configuration make it particularly appealing for mobile/maritime satellite communications, remote sensing/tracking, telemetry, and wireless local-area network applications [3]. In [4,5], we presented very low-mass SBF antennas with excellent performance, such as a high gain and wide bandwidth. In this work, our goal is to further enhance the SBF antenna’s gain and bandwidth without sacrificing other crucial performance factors like the cross-polarization ratio. In doing this, we aim to make it more appealing for the targeted applications.

Several gain-enhancing techniques for many antennas that have been presented in the literature can also be applied to the SBF antenna. The first technique involves the design of antenna arrays such as the horn antenna array [6,7] and patch antenna array [8,9]. Combining the performance of the individual elements results in overall increased gain and directivity. While this is a very effective way of achieving higher gain, some designs require a multilayer design [9] and a very sophisticated feed network, thereby increasing the cost and complexity of the antenna. This technique can be challenging for applications with limited antenna space, since multiple antennas increase the final size.

Another common technique involves increasing aperture efficiency by manipulating the aperture phase. This has been done in the literature by employing metasurfaces [10,11] such as electro bandgap (EBG) materials [12,13], partially reflective surfaces (PRS), or a meta surface lens [14,15] over the opening of the antenna’s aperture. Although metamaterials have been effective in increasing horn antenna gain, their complex design and narrow bandwidth are downsides. The metasurface’s resonant structure limits the bandwidth of the antenna, as it determines the band of operation which is usually very narrow.

Increasing the antenna’s physical aperture size [16,17] is the third gain enhancement technique that has been widely used and can be applied to SBF antennas. The aperture size can be increased by flaring the opening of the antenna, like the design of a horn antenna, which is essentially a flared waveguide antenna [18]. Signals traveling through the waveguide experience an abrupt change in impedance from the waveguide to free space, which is approximately 377 Ω. As a result of this change, signals are reflected along the waveguide as standing waves, similar to having poor matches at the end of coaxial or other wire-based transmission lines. The waveguide is usually flared or tapered out to get around this problem. This has the effect of allowing the impedance of the waveguide to gradually change to that of free space. It functions as a progressive matching transformer, minimizing the reflected signal and thereby increasing the efficiency of the antenna. 

The flaring angle has a great effect on the antenna gain and directivity. As the flaring angle and the aperture size increase, the gain increases. However, there is an optimal angle, beyond which the gain starts to decrease with an increasing angle of flare. This is because of the increasing phase error (phase difference between the edges and center point), which cancels out the gain [19]. Because the flaring angle required to achieve higher gain and bandwidth is minimal, the increase in the size of the antenna is not very significant. This is in contrast to the metasurface technique, which makes the flaring technique appealing for the SBF antenna. For some antenna designs, such as the patch antenna with a W-shaped ground plane [19], the impedance bandwidth increases with the flaring angle as a result of improved impedance matching.

The objective of this paper is to improve the gain and impedance bandwidth of the SBF antenna design by integrating two design principles: introducing a flare at the rim and adding an inductive iris at the waveguide feed aperture. To describe the performance, a simulation study was conducted, and the principles were verified by fabricating a prototype using a 3D printer. The methods examined in this article have the potential to be applied to similar types of antennas. Section 2 describes the geometry of the SBF antenna, and the study on the effect of the flaring angle and the rim height on the SBF antenna is presented. The design of the flared SBF antenna with an inductive iris and the parametric studies are presented in Section 3. In Section 4, we provide the measurement results for the fabricated SBF antenna, while the conclusion and future work are presented in Section 5.

## 2. Flared SBF Antenna

### 2.1. Antenna Configuration

**Figure 1:**
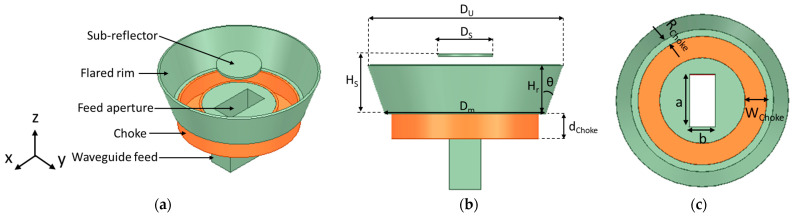
The geometry of the flared SBF antenna. (**a**) Isometric view, (**b**) Front view, and (**c**) Top view.

This study aims to further increase the gain and bandwidth of the antenna presented in [4,20] while maintaining its cross-polarization ratio. The flared SBF antenna shown in Figure 1 consists of several parts and is fed by a waveguide feed because of its high gain and high-power handling abilities. The conventional SBF antenna consists of only four parts: the main reflector, the rim, the sub-reflector, and the feed antenna. The choke portion was included in the work presented in [4,20] as a way of increasing the gain. In this study, to further increase the gain, we decided to flare the upper diameter of the antenna’s rim, similar to the design of the horn antenna. Like the horn antenna, the flare angle has a significant effect on the gain. As the flare angle increases, the gain also increases until it reaches the optimum flare angle. Although much of the research that has been conducted shows that antenna flaring does not affect the impedance bandwidth, we observed a significant improvement in the impedance bandwidth of the proposed antenna design when the rim is flared as a result of resonance merging. Several parametric studies have been conducted to find the optimum flare angle and rim height by varying the flare angle (θf) and rim height (Hr) and examining the behavior of the gain and the bandwidth.

The SBF antenna’s main reflector, choke, and rim are fabricated using 3D printing technology, after which the entire surface is then metalized using a conductive spray. To model this in HFSS, the entire structure is first designed with a PLA plastic material (ε_r_ = 2.1, tanδ = 0.005), and an impedance boundary condition is then applied all over the surface of the structure to simulate the surface resistance of the silver metal coating (0.05 Ω/sq). The sub-reflector is made of aluminum metal; therefore, it is designed using the perfect electric conductor (PEC) material, while the waveguide feed is modeled as an open-ended waveguide structure (PEC) with a wave port feed. The optimized dimensions of the flared SBF antenna are provided in Table 1, and are also expressed in terms of the free space wavelength (λ). λ is equal to c/f, where c = 3 × 10^8^ is the speed of light, and f = 5.5 GHz is the operating frequency.

### 2.2. Numerical Analysis

The use of rim flaring to increase gain and its impact on the impedance bandwidth and cross-polarization ratio are covered in detail in this section. The ANSYS Electronics Desktop was used for carrying out all the simulations for this paper. 

#### 2.2.1. Effect of the Flaring Angle

During the first parametric study, the flaring angle (θf) was increased from 0° to 40° in increments of 5° while keeping all other variables constant. This was done to investigate the effect of increasing the flaring angle. The upper diameter of the rim (D_U_) can be expressed in terms of the flaring angle as D_m_ + 2. Hr. tan θf, where D_m_ is the diameter of the main reflector and the lower diameter of the rim, and Hr is the height of the rim. All the simulations in this section used Hr = 0.6 λ (where λ is the wavelength of the SBF antenna in free space), which was the optimized rim height from the unflared SBF antenna study. The case where θf = 0° gives the results of the unflared antenna, which were compared to the results obtained at different flaring angles.

**Figure 2:**
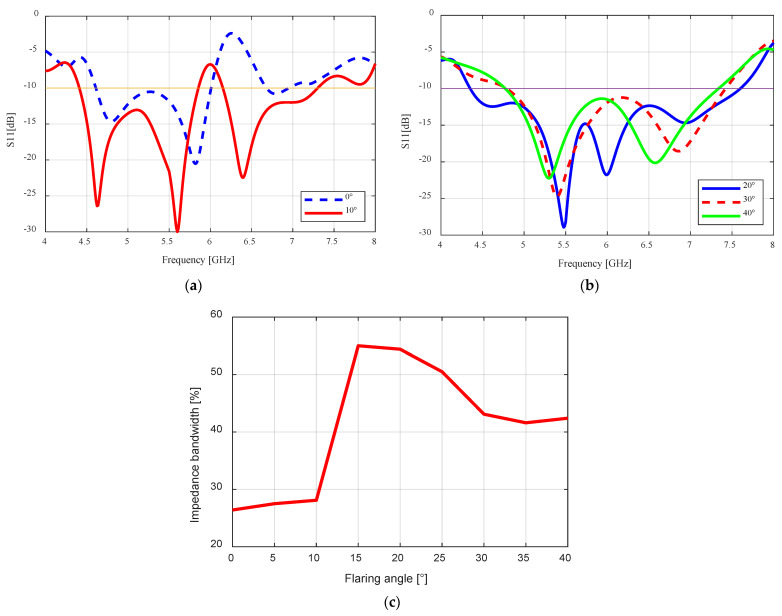
Flared SBF antenna at different flaring angles: (**a**) Reflection coefficient plot for flaring angles 0° and 10° as a function of frequency; (**b**) Reflection coefficient plot for flaring angles 20° to 40° as a function of frequency; (**c**) Impedance bandwidth as a function of the flaring angle.

The reflection coefficient (S11) results are presented in Figure 2a–c. As the flaring angle increased from 0–10°, there was no significant change in the impedance bandwidth (Figure 2a), as it only increased from 26.4% (4.61–6.01 GHz) to 28.1% (5.85–4.41 GHz), respectively. The major change was observed when the flaring angle was increased to 15° (Figure 2c) and the highest impedance bandwidth of 55% (4.36–7.67 GHz) was obtained, which is more than double the result of the unflared case (26.4%). This bandwidth increase is a result of the coupling of the two resonances seen at 5.8 GHz and 6.8 GHz (1 GHz difference) for the 0° flaring angle (Figure 2a). Flaring the antenna to 15° brought the resonances together to 5.5 GHz and 6 GHz (0.5 GHz difference), causing them to overlap, thereby resulting in the larger impedance bandwidth observed. We observe that when we increased the flaring angle further to 40°, the bandwidth decreased to 42.4% (4.77–7.34 GHz) as illustrated by Figure 2c.

**Figure 3:**
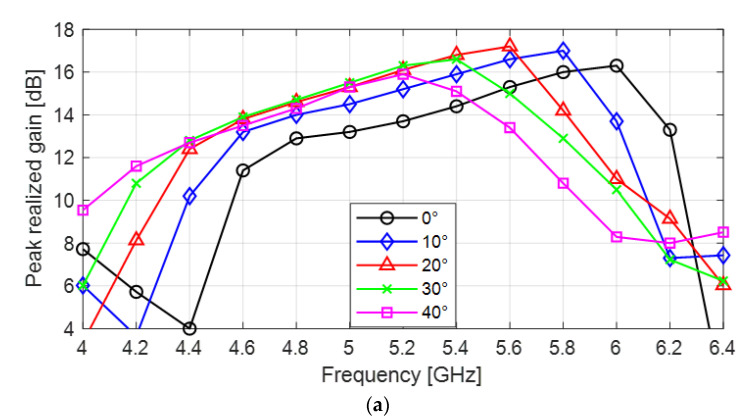

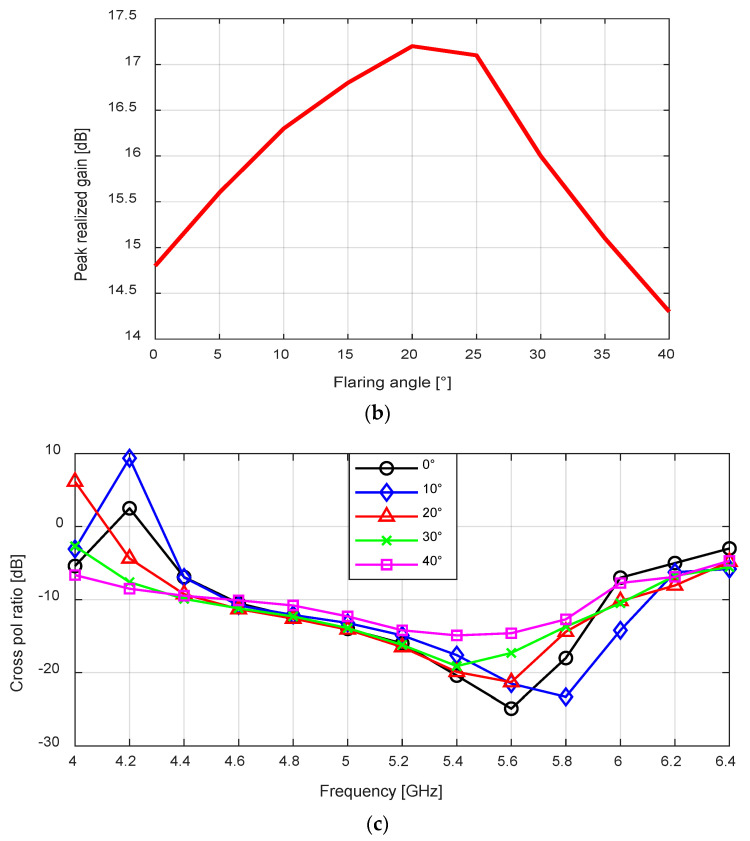
Flared SBF antenna at different flaring angles: (**a**) Peak realized gain plot for flaring angles 0° to 40° as a function of frequency; (**b**) Peak realized gain plot as a function of the flaring angle; (**c**) Cross polarization ratio plot for flaring angles 0° to 40° as a function of frequency.

The simulated gain and cross-polarization ratio results for the flared SBF antenna are given in Figure 3. Figure 3a shows the peak realized gain plot as a function of frequency for flaring angles 0–40°, while the peak realized gain trend with increasing flaring angle at a frequency of 5.5 GHz is shown in Figure 2b. As the flaring angle increased from 0° to 20°, the peak realized gain increased from 14.8 dBi to 17.2 dBi (a 2.4 dB increase) at 5.5 GHz (center frequency). This increase in gain, as discussed in previous sections, is a result of having a better impedance matching at the edge of the antenna’s aperture due to reduced back reflection of waves. From the plots in Figure 2, it can be observed that increasing the flaring angle further from 20° to 40° does not improve the gain. There was a small gain drop from 17.2 dBi to 17.1 dBi when the flaring angle was increased to 25°, then the realized gain can be seen to plummet when the flaring angle was increased to 40° from 17.1 dBi to 14.3 dBi. This is mostly due to an increase in the phase error due to over-flaring. The 20° flaring angle also produced the widest gain bandwidth of 1.1 GHz or 21% (4.7 to 5.8 GHz) using the 3 dB gain definition. From these observations, it can be deduced that the optimum flaring angle to achieve the highest gain for this design is 20°.

Figure 3c shows the worst-case cross-polarization ratio at θ = 45°. The results show that increasing the flaring angle from 0° to 40° generally increases the cross-polarization ratio. The minimum cross-polarization ratio for the unflared antenna is −24 dB at 5.6 GHz, while the minimum cross-polarization ratios for the flared antenna are −23.27 dB (5.8 GHz), −21.32 dB (5.6 GHz), −19.43 dB (5.4 GHz), and −14.84 dB (5.4 GHz) at θf = 10°, 20°, 30°, and 40°, respectively. Although this study shows an increase in the overall cross-polarization ratio, we found that at lower flaring angles such as 10° and 20°, the increase is not as significant as those of higher flaring angles (30° and 40°). 

The performance of the flared antenna is summarized in Table 2. The impedance bandwidth increased as the flaring angle increased, having the highest value at θf = 15° and then this value started to decrease gradually. Gain improvement was also observed with increasing flaring angle until θf = 20°. The cross-polarization ratio decreased as the flare angle was increased. From the simulation results and observations, the optimum flaring angle for this design is at θf = 20°. Although the highest bandwidth was obtained at θf = 15°, the obtained gain at the same angle is ~0.5 dB less than that of θf = 20°, while the bandwidth at θf = 20° is lower than the highest bandwidth by just 0.6% (0.06 GHz). The difference in impedance bandwidth is very minimal compared to the gain difference, therefore we chose the flared SBF antenna with θf = 20° as the optimum design.

#### 2.2.2. Effect of the Rim Height

This parametric study focuses on the effect of the rim height on the gain and impedance bandwidth of the flared SBF antenna. We decided to keep the flaring angle for this section constant at θf = 20°. We chose this flaring angle because it provided the highest gain and wide bandwidth in the previous section. It is important to note that while the flaring angle is kept constant, D_U_ (upper diameter of the rim) still changes because of the changing height. 

**Figure 4:**
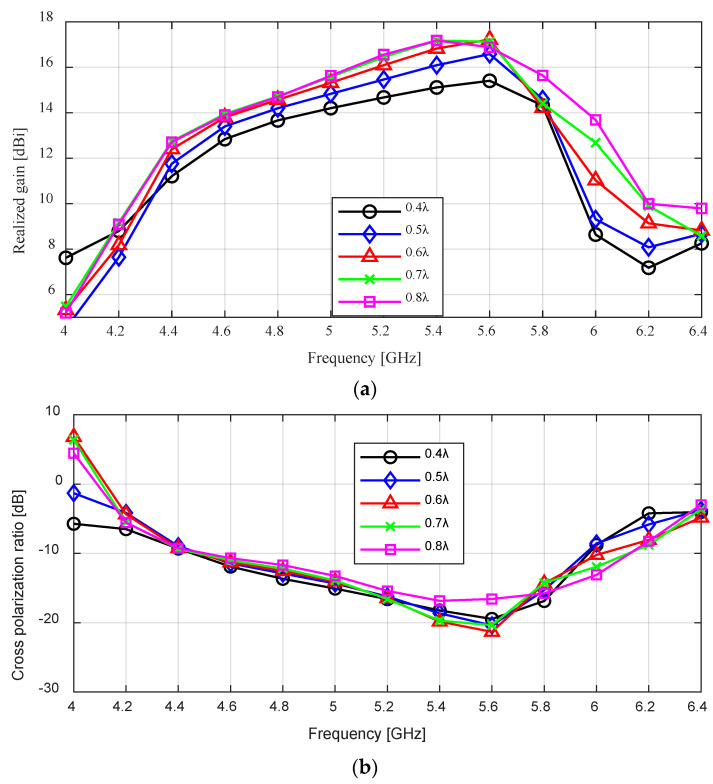

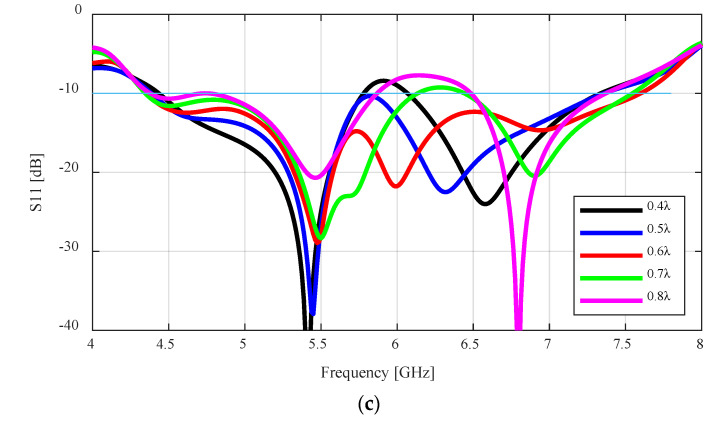
Effect of height variation as a function of frequency. (**a**) Realized gain; (**b**) Cross-polarization ratio; (**c**) Reflection coefficient.

To study the effect of the rim height, we varied it from 0.4 λ to 0.8 λ with a step of 0.1 λ. The simulated realized gain results for different rim heights are shown in Figure 4a. As the rim height increased from 0.4 λ to 0.6 λ, the peak realized gain can be seen to increase from 15.41 dBi to 17.22 dBi (an increase of 1.81 dB) at 5.6 GHz; the gain at 5.5 GHz also improved from 15.27 dBi to 17.02 dBi (a 1.75 increase). There was no significant change in the realized gain when the rim height was increased further to 0.8 λ. While the cross-polarization ratio does not change significantly as the rim height is increased, we can see in Figure 4b that the minimum cross-polarization ratio of 21.32 dB was obtained at 0.6 λ.

The effect of varying the height of the rim on the reflection coefficient (S11) can be seen in Figure 4c. Increasing the height of the rim from 0.4 λ to 0.6 λ lowered the reflection coefficient result of the antenna, thereby increasing the impedance bandwidth from 26.4% (4.43–5.78 GHz) to 55% (4.35–7.60 GHz). This value dropped to 29.1% (4.38–5.87 GHz) as the height is increased further to 0.8 λ. This shows that the rim’s height also affects impedance matching.

Table 3 summarizes the effect of the rim’s height on the flared SBF antenna. From this study, we observe that varying the height of the SBF antenna’s rim while keeping the flaring angle and other dimensions constant affects the gain and impedance bandwidth result, while the cross-polarization ratio was not significantly affected. The highest gain, widest impedance bandwidth, and lowest cross-polarization ratio were obtained at Hr = 0.6 λ. The performance of the antenna was worse at other Hr values.

## 3. Flared SBF Antenna with Iris

**Figure 5:**
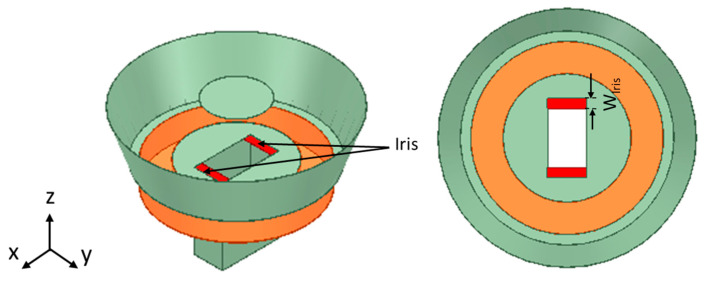
The geometry of the flared SBF antenna with an iris.

To further improve the reflection coefficient (S11) of the flared SBF antenna, the length of the waveguide feed aperture (a = 40.387 mm) was shortened on both sides by adding thin metal strips as shown in Figure 5. These metal strips are usually referred to as a waveguide iris. The waveguide iris can either model a shunt capacitance or inductance, depending on whether it is located in either the transverse plane of the electric field or the magnetic field [21]. In our case, the iris was placed in the magnetic field, hence it created an inductive element that can provide the necessary matching for the waveguide’s characteristic impedance. Improved impedance matching lowers the S11 result, thereby increasing the impedance bandwidth. The flaring angle and the rim height were kept constant at θf = 20° and Hr = 0.6 λ, as these produced optimized gain, bandwidth, and cross-polarization results in the previous section.

A parametric study was carried out in this section to investigate the effect of the iris on the reflection coefficient (impedance bandwidth) and the gain of the flared SBF antenna. The length of the iris is the same as the width of the waveguide feed (b = 20.193), while the width of the iris is varied from 1 mm to 5 mm with a step of 1 mm to determine the best value for optimal performance. The results are compared to the design without the iris (0 mm iris width).

**Figure 6:**
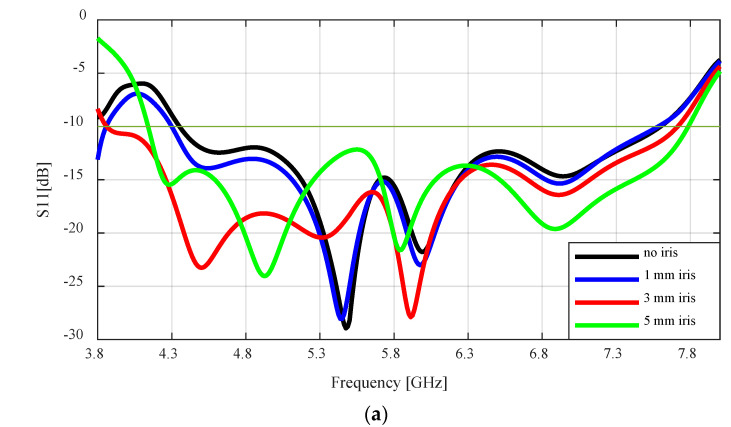

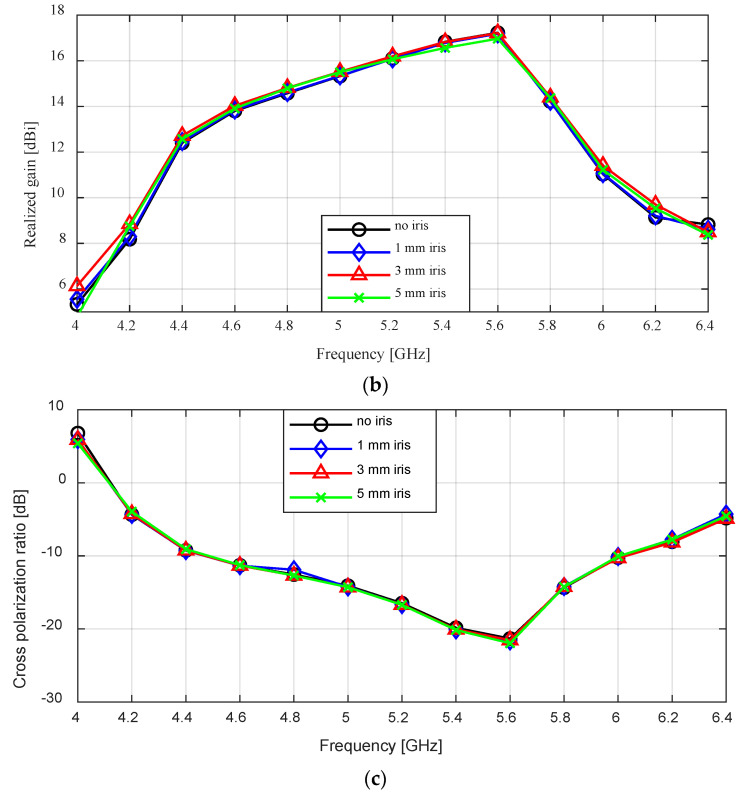
Flared SBF antenna with different iris widths: (**a**) Reflection coefficient as a function of frequency; (**b**) Peak realized gain as a function of frequency; (**c**) Cross-polarization ratio as a function of frequency.

The effect of the inductive iris on the reflection coefficient (S11), impedance bandwidth, and peak realized gain are provided in Figure 6. Figure 6a shows the reflection coefficient plots for different iris widths (0 to 5 mm) at a 20° flaring angle. As the width of the iris is increased from 0–3 mm, the frequency bandwidth for S11 < −10 dB increased from 3.3 GHz or 55% (4.3–7.6 GHz) to 3.85 GHz or 66.7% (3.85–7.7 GHz). As the iris width is increased from 3–5 mm, the bandwidth starts to decrease from 3.85–3.7 GHz 62.2% (4.1–7.8). The 3 mm iris width provides the best impedance matching between the waveguide feed and the SBF antenna as it produces the widest bandwidth. 

Figure 6b,c shows the effect of the iris on the realized gain and the cross-polarization ratio of the flared SBF antenna. As can be seen in the figure, introducing the iris does not affect the gain of the antenna and the cross-polarization ratio of the flared SBF antenna. The peak realized gain and the minimum cross-polarization ratio for all cases were ~17 dBi at 5.6 GHz and −21 dB at 5.6 GHz, respectively. This is expected as the purpose of the iris is to improve the impedance matching.

## 4. Flared SBF Antenna with a Superstrate Lid 

Some of the previous designs of waveguide-fed SBF antennas have utilized foam materials to support the sub-reflector at the required height from the main reflector. Although the foam material has been useful for prototyping, it is not strong enough to keep the sub-reflector permanently in place and could potentially be modified when installed for a practical application. For example, the foam could be pushed downward into the main reflector, and the optimized position of the sub-reflector would be compromised. To solve this issue, we developed a thin plastic-based superstrate that is rigid enough to maintain the position of the sub-reflector with minimal effect on the antenna’s performance. Furthermore, the superstrate can be easily 3D printed. 

It is important to judiciously choose the right thickness and material of the superstrate to minimize the reflections that can negatively affect the antenna’s performance. We carried out simulation studies to compare the performance of the antenna with and without the superstrate to ensure that excellent performance is maintained.

**Figure 7:**
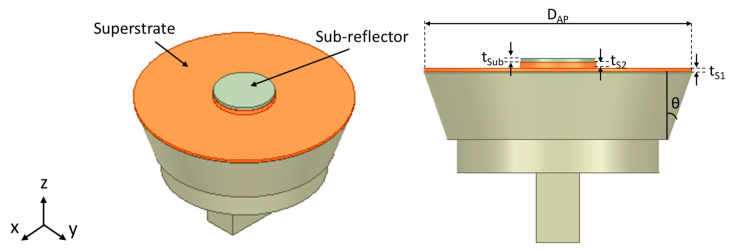
Flared SBF antenna with a dielectric superstrate cover.

The geometry of the flared SBF antenna with the substrate lid is shown in Figure 7. The lid consists of two parts that are connected together, a thin disk with a hole and a rim surrounding the hole on which the sub-reflector is placed. The dimensions t_S1_, t_S2_ and t_Sub_ refer to the thickness of the disk, the thickness of the lid’s rim, and the thickness of the sub-reflector. The distance between the rim and the sub-reflector (Hr − Hs = 5.45 mm) is equal to t_S1_ + t_S2_, t_Sub_ = 2 mm, while the diameter of the disk’s hole is equal to 37.5 mm. 

For the simulation, we studied the effects of the lid on the behavior of the antenna by modeling it with two plastic materials with different dielectric constants. The first is the polylactic (PLA) plastic with a dielectric constant of 2.1, while the second plastic is the acrylonitrile butadiene styrene (ABS) plastic with a dielectric constant of 3.0. The thickness of the lid’s disk (t_S1_) was also varied from 1–4 mm and studied to obtain the best design performance. 

**Figure 8:**
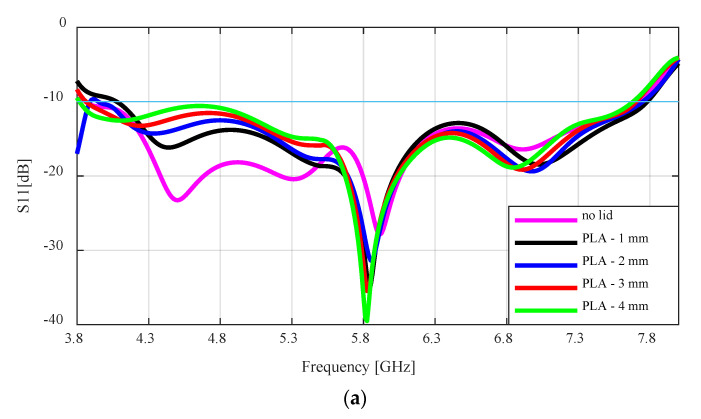

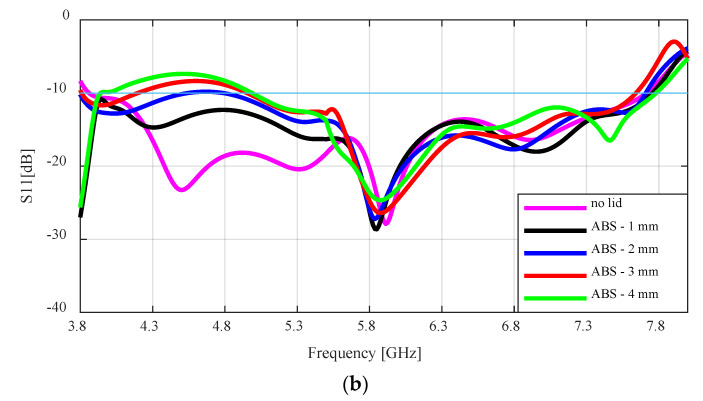
Simulated S11 result of the flared SBF antenna with: (**a**) PLA lid; (**b**) ABS lid.

Figure 8a,b gives the simulated S11 result of the flared antenna. From both plots, it can be seen that the antenna with the lid raised the S11 result compared to the antenna with no lid. The effect of the PLA lid with the lower dielectric constant on the antenna is not as significant as that of the ABS lid. While the S11 result for the PLA lid is raised between 4.3–5.6 GHz, the value does not go above −10 dB, therefore the bandwidth does not change much. All the simulated impedance bandwidths were between 62.2% to 67.8% for 4 mm to 1 mm lid thickness, respectively.

As the thickness of the ABS lid increases above 1 mm, the S11 result is raised above −10 dB at the lower frequencies, which drastically reduced the impedance bandwidth. The bandwidth for the antenna with no lid is 66.7%, while the antenna with the ABS lid of 1 mm, 2 mm, 3 mm, and 4 mm had impedance bandwidths of 66.4%, 46.4%, 43.2%, and 45.7%, respectively.

**Figure 9:**
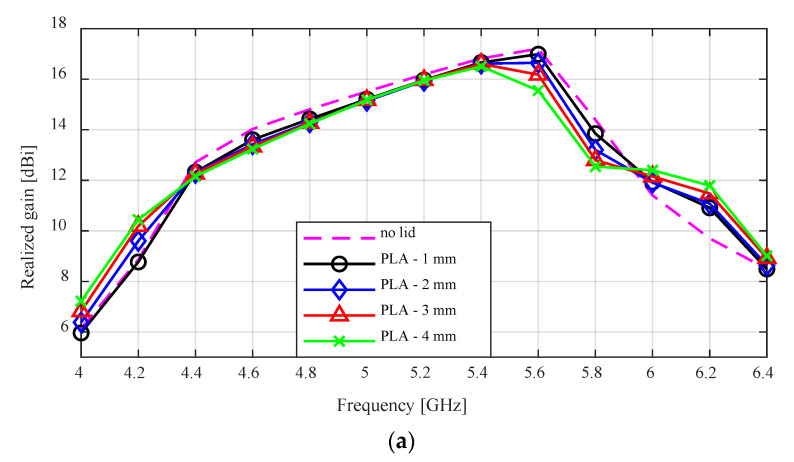

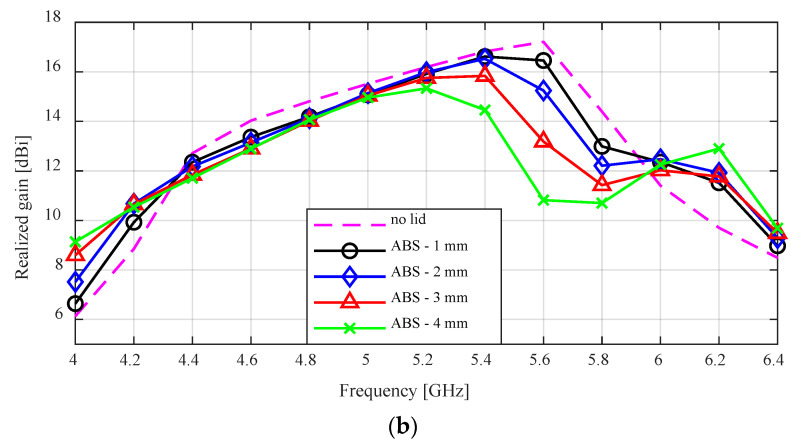
Simulated peak gain result for the flared SBF antenna with: (**a**) PLA lid; (**b**) ABS lid.

The peak realized gain for the SBF antenna with the PLA and ABS lids are compared to the antenna without the lid in Figure 9. There is a general trend of the gain reducing with increasing lid thickness. However, the gain loss experienced by the PLA lid is not as high as that of the ABS with the higher dielectric constant. This gain loss is most noticeable at 5.6 GHz and with a lid thickness of 4 mm. For example, at 5.6 GHz, the lidless antenna has a peak gain of 17.02 dBi, while the PLA and ABS lids with a lid thickness of 4 mm have peak gains of 15.7 dBi and 10.8 dBi, respectively. The gain loss is very minimal for the 1 mm lid thickness. The antennas with the 1 mm lid thickness at 5.6 GHz had peak gains of 17.01 dBi and 16.8 dBi for the PLA and ABS lids, respectively.

**Figure 10:**
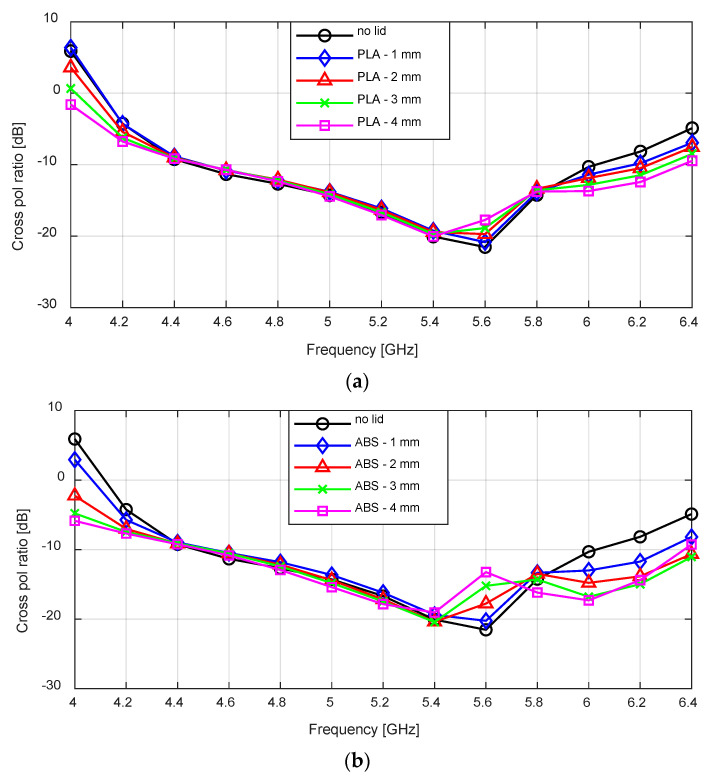
Simulated cross-polarization ratio for the flared SBF antenna with: (**a**) PLA lid; (**b**) ABS lid.

The worst case cross-polarization ratio results for the flared antenna with the lids are shown in Figure 10. For the PLA lid, there is no observable difference between the results of the no lid case and the varying lid thickness cases between 4.4 to 5.4 GHz. Between 5.4 to 5.8 GHz, the minimum cross-polarization increased from −21 dB to −17 dB as the lid thickness increased from 1–4 mm. This is the same case for the ABS lid between 4.4 to 5.4 GHz, but the increase in the cross-polarization ratio is higher as it increased to −13 dB for a 4 mm lid thickness.

## 5. Fabrication and Testing

For the fabrication of the flared SBF antenna, we employed the fused deposition modeling (FDM) 3D printing technique to achieve a low-mass design and reduce production cost and time. The flared SBF antenna was fabricated at the University of Manitoba’s ECE machine shop using the Anykubic 3D-printer and polylactic acid (PLA) thermoplastic filament. A polishing post-processing step was performed to eliminate the ridges that formed on the antenna surface due to the 3D-printing layer-by-layer process. This was achieved by applying XTC-3D coating, which is a liquid that consists of a resin part and a hardener part that are mixed together. When a thin layer of this resin-based liquid is applied to the inner surface of a 3D-printed prototype, it fills the ridges, smoothens the surface, and hardens into a resistant coating.

**Figure 11:**
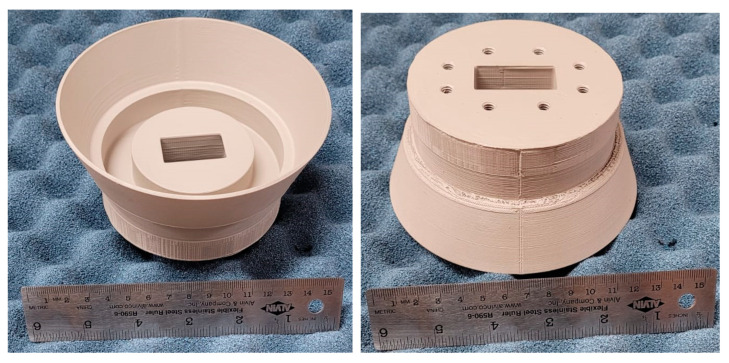
Top (**left**) and bottom (**right**) views of the fabricated flared SBF antenna.

After the polishing step, the antenna was coated with MG chemical’s silver conductive coating, which is required to create a conductive surface. The top and bottom views of the conductive 3D-printed flared SBF antenna are provided in Figure 11. 

**Figure 12:**
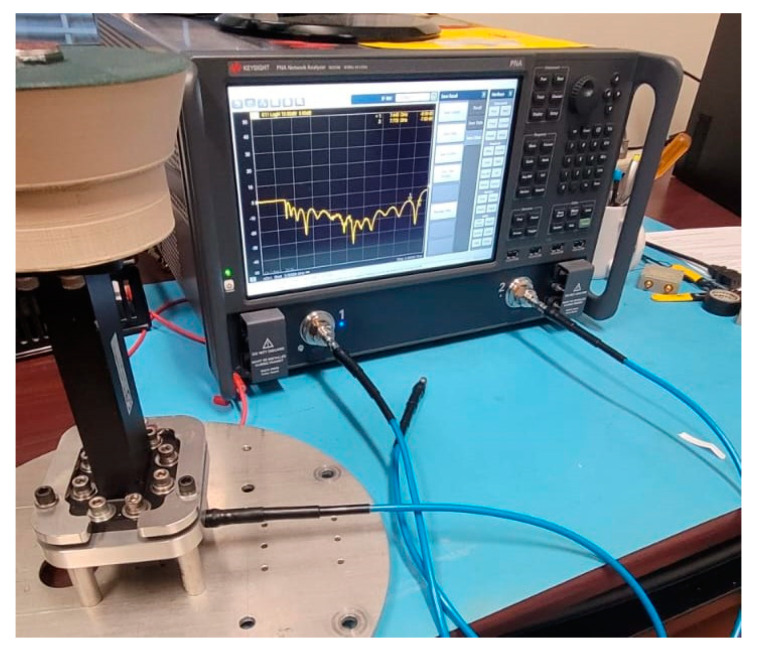
The fully assembled flared SBF antenna connected to the VNA.

**Figure 13:**
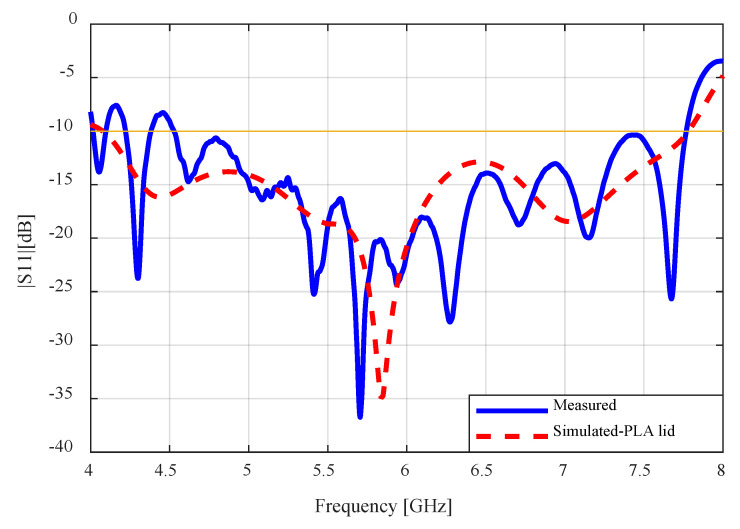
Measured and simulated S11 results of the flared SBF antenna.

The fully assembled antenna consisting of the WR-159 waveguide feed, the PLA cover, and the sub-reflector is shown in Figure 12. In the figure, the waveguide port is connected to the first port of the Keysight PNA Network Analyzer (N5224B) to measure the S-parameter. The measured S11 result is compared to the simulated result in Figure 13. There is very close agreement between the patterns of both plots. The fluctuation in the measured results comes mostly from the waveguide feed. The impedance bandwidth of the fabricated antenna was measured to be 53.7% (4.5–7.8), which is less than that of the simulated result (62.2%).

**Figure 14:**
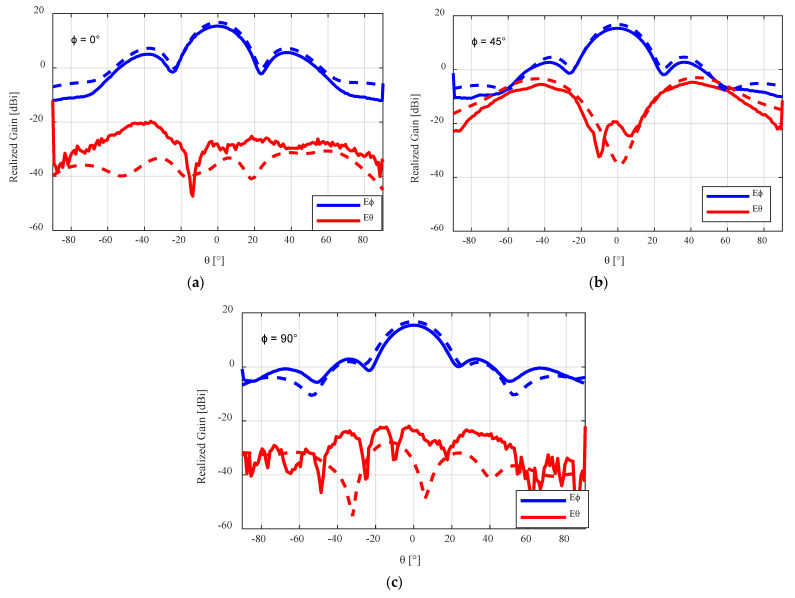
Measured radiation pattern results for the flared 3D-printed SBF antenna with the PLA lid at (**a**) φ = 0°, (**b**) φ = 45°, and (**c**) φ = 90°. The dashed lines represent the simulated results, while the solid lines represent the measured results.

The far-field radiation pattern of the antenna was also measured in the anechoic chamber at the University of Manitoba’s antenna laboratory. The radiation pattern results of the flared SBF antenna at ϕ = 0°, 45°, and 90° are shown in Figure 14. The simulated peak gain (which considers the 3D-printed materials used) was 16.78 dBi, while the measured peak realized gain was 15.4 dBi, indicating a gain loss of approximately 1.36 dB. For the worst case cross-polarization analysis, the measured result at φ = 45°, was 20.77 dB (15.34–(−5.43)), which is very close in agreement with the simulated result of 20.15 dB (16.78–(−3.37)). While the other radiation characteristics of the fabricated antenna closely match the simulated results, the gain loss was more than anticipated.

**Figure 15:**
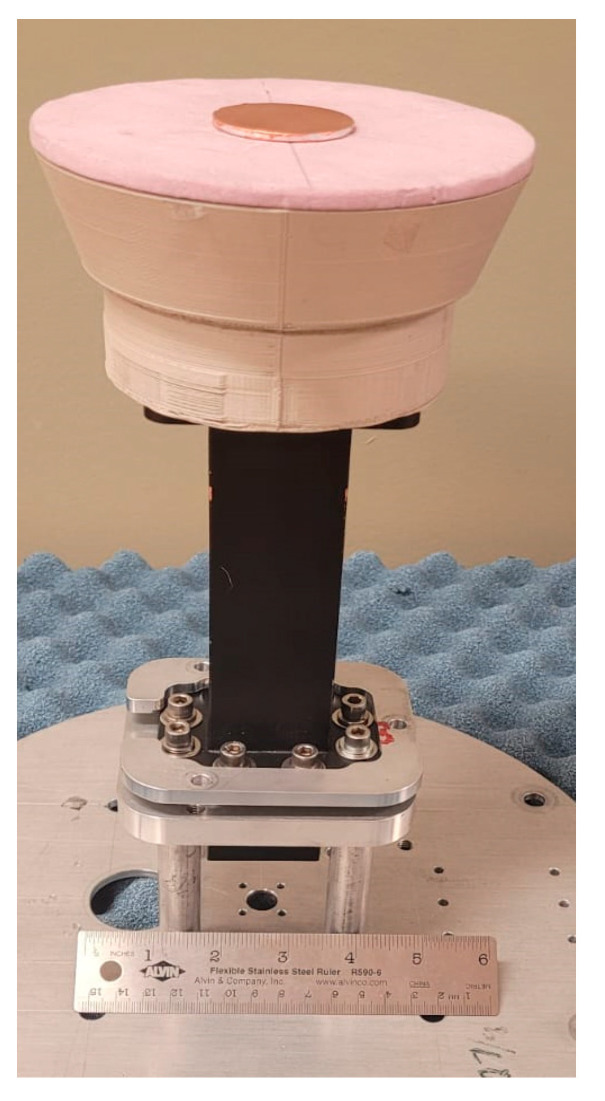
The flared SBF antenna with the foam lid.

To determine the source of this error, a piece of foam with a known dielectric constant of 1.05 was used to replace the PLA lid in order to test if the antenna’s gain loss was due to the PLA lid, as shown in Figure 15. The thickness of the foam was designed to be the same dimension as the difference in height between the rim and the sub-reflector (Hs − Hr = 38.181 − 32.727 = 5.454 mm). To compare with the measured results, further simulation analyses using the foam material were performed on the flared SBF antenna. 

**Figure 16:**
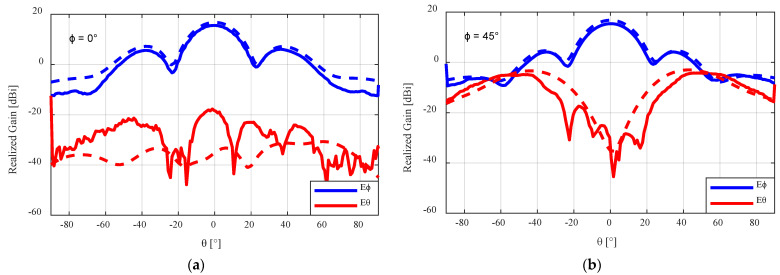

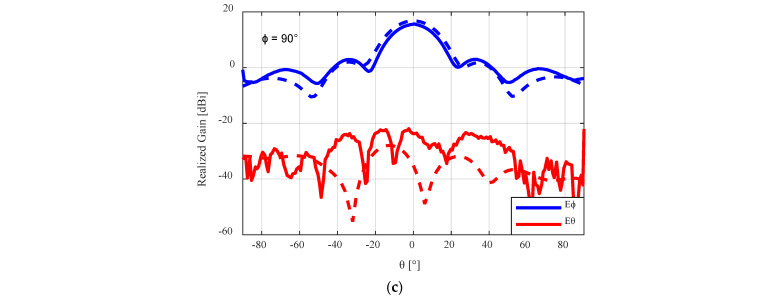
Measured radiation pattern results for the flared 3D-printed SBF antenna with the foam lid at (**a**) φ = 0°, (**b**) φ = 45°, and (**c**) φ = 90°. The dashed lines represent the simulated results, while the solid lines represent the measured results.

The measured and simulated results of the flared SBF antenna with the foam lid are shown in Figure 16. The measured peak gain increased from 15.4 dBi to 15.7 dBi when compared to that of the results of the antenna with the PLA lid. A gain loss of about 1 dB was experienced compared to the simulated peak gain result of 16.74 dBi. Accounting for about 0.6 dB loss, which is expected from imperfect connections between the antenna and the waveguide feed and also from the connected cables, the remaining 0.4 dB can be attributed to the additive manufacturing process. The simulated and measured worst case cross polarization ratio for the flared SBF antenna with the foam lid, which were 20.54 dB, and 19.95 dB, respectively, are very close in agreement.

To demonstrate the superiority of 3D printing over the conventional milling fabrication process, we measured the weight of a PLA (thermoplastic) antenna and an aluminum prototype. The weight of the PLA antenna was 252 g, while that of the aluminum prototype was 576.5 g. This implies a 56.3% reduction in mass for the PLA antenna, which is crucial in applications like satellite communications and remote sensing where increased mass can lead to a significant increase in cost.

## 6. Conclusions

The objective of the paper was to design an improved low-mass short backfire antenna for space and remote sensing applications. A combination of techniques was presented in this paper to improve the gain, bandwidth, and structural integrity of the waveguide-fed SBF antenna while reducing the total mass. Gain and bandwidth enhancement was achieved by employing the aperture flaring technique, which involved flaring the rim of the SBF antenna to an optimal angle of 20°. After optimizing both the flaring angle and the rim height, the gain and bandwidth improved from 14.8 dBi to 17.2 dBi and from 26.4% to 54.4%, respectively, while having no significant effect on the cross-polarization ratio. The inductive iris was then used to further improve the impedance matching and the bandwidth of the antenna from 54.4% to 66.7%, while the gain and cross-polarization remained constant. 

Due to the low structural integrity of the foam lid that was used in the previous SBF antenna designs, a 3D-printed plastic lid that is stronger than the foam material was utilized. Both lids were then tested to compare their performance. We observed that the gain loss of the antenna with the plastic lid was a little higher than the gain loss of the antenna with the foam lid. This higher gain loss can be attributed to the higher dielectric constant of the plastic material. Finally, the antenna was fabricated with plastics by employing the additive manufacturing technique (3D printing) to significantly reduce the mass of the antenna. The measured results of the 3D-printed flared SBF antenna were in good agreement with the simulated results. However, some additional gain loss of about 0.4 dB was measured due to the increased surface impedance as a result of the additive manufacturing process.

The flared SBF antenna’s exceptional performance was demonstrated through comparison with other antenna designs (SBF and horn antennas) that operate within different microwave frequency ranges listed in Table 4. This table gives the frequency range of operation along with a comparison of the antenna designs’ gain and impedance bandwidth. The weight and cross-polarization ratio of these designs were not disclosed, hence these characteristics were not included. The gain values of the flared antenna are similar to those of the antennas mentioned in [20,22,23]. However, the impedance bandwidth of the flared antenna is much wider than that of the above-mentioned antennas. This implies that the flared antenna can operate over a broader range of frequencies without significant changes in its performance, making it a more versatile option for similar applications. When compared to the antennas described in [24,25,26,27], the flared SBF antenna performs better in terms of both gain and impedance bandwidth. The flared SBF was also compared to the standard gain C-band horn antenna [28], which is commonly used for the same applications as the SBF antenna. It can be seen from the table that the performance of the flared SBF antenna is superior to that of the standard gain horn antenna, having a higher gain and much wider impedance bandwidth. Therefore, the flared SBF antenna can be easily used as a better replacement for the horn antenna in satellite communication and remote sensing applications.

The research presented in this paper showcases the potential of additive manufacturing for producing RF components, specifically antennas designed for space and remote sensing applications. This potential goes beyond reducing the mass, as demonstrated in the study, and includes the ability to manufacture complex designs that would have been difficult to achieve with traditional subtractive manufacturing processes. Additionally, this technique can significantly reduce the cost and time needed for production. The results of this work can serve as a useful starting point for optimizing similar antenna types. The proposed enhanced low-mass short backfire antenna outperforms the C-band standard gain horn antenna and many other short backfire antennas found in the literature in terms of impedance bandwidth and/or gain performance. Hence, it can be used as a superior substitute in many applications.

## Figures and Tables

**Table 1:** Simulated dimensions of the Flared SBF antenna.

Parameter	Description	Value (mm)
Dm	Diameter of the main reflector	110.00 (2.02 λ)
D_U_	Upper diameter of the rim	Dm + 2. Hr. tan θ
D_S_	Diameter of the sub-reflector	38.181 (0.70 λ)
Hr	Height of the rim	32.727 (0.60 λ)
H_S_	Height of the sub-reflector	38.181 (0.70 λ)
Rchoke	Distance between the rim and outer diameter of the choke	5.455 (0.01 λ)
dchoke	Depth of the choke	16.064 (0.29 λ)
Wchoke	Width of the choke	16.064 (0.29 λ)
a	Length of the waveguide	40.387
b	Width of the waveguide	20.193

**Table 2:** Performance comparison of the flared SBF antenna with different flaring angles (θf).

Hr	θf (°)	D_U_ (mm)	Realized Gain @ 5.5 GHz (dBi)	Imp. BW (%)	Min. Cross-Pol. Ratio (dB)
0.6 λ	0	110	14.8	26.4	−24.00
	10	121.5	16.3	28.1	−23.27
	15	127.5	16.7	55.0	−22.15
	20	133.8	17.2	54.4	−21.32
	30	147.8	16.0	43.1	−19.43
	40	165	14.3	42.4	−14.84

**Table 3:** Performance comparison of the flared SBF antenna with different rim heights (Hr).

θf	Hr (λ)	D_U_ (mm)	Peak Gain (dBi)	Gain @ 5.5 GHz (dBi)	Imp. BW (%)	Min. Cross-Pol. Ratio (dB)
20°	0.4	125.9	15.41	15.27	26.4	19.73
	0.5	129.9	16.58	16.34	50.6	20.4
	0.6	133.8	17.22	17.02	55.0	21.32
	0.7	137.8	17.18	17.04	33.0	20.35
	0.8	141.8	17.18	17.15	29.1	16.9

**Table 4:** A comparison of the proposed flared SBF antenna with other SBF and horn antennas.

Reference	Description	Frequency Range (GHz)	Impedance Bandwidth (%)	Measured Peak Gain (dBi)
This work	Flared SBF antenna	4–7	53.7	15.7
[20]	Waveguide-fed SBF antenna	4–7	27.3	15.7
[22]	Patch-Fed Short Backfire Antenna	2–2.5	15	15.2
[23]	3D-printed low-cost choke corrugated Gaussian profile horn antenna for Ka-band	26.5–40	14.3	15.2
[24]	Substrate Integrated Waveguide H-plane Horn Antenna	85–103	19.6	14.5
[25]	Metamaterial-enabled short backfire antenna	1.1–1.75	10	14.1–15.7
[26]	K-Band Horn Antenna	17.5–20.5	16.2	14.5
[27]	Horn antenna with integrated metamaterial for beam steering	10–11.5	4.7	13.3
[28]	Standard Gain C-band Horn Antenna	4–7	36.0	15.0

## Data Availability

Data available within the article.

## References

[1] Kirov G.S. (2009). Design of short backfire antennas. IEEE Antennas Propag. Mag..

[2] Ehrenspeck H.W. (1965). The Short-Backfire Antenna. Proc. IEEE.

[3] Ehrenspeck H.W. (1967). High-Gain UHF Backfire Antenna for Communications, Telemetry, and Radio Astronomy.

[4] Aragbaiye Y.M., Mansoori A., Shafai C., Isleifson D. Implementing a Prototype of a Short-Backfire Antenna Using Additive Manufacturing. Proceedings of the 2022 IEEE International Conference on Wireless for Space and Extreme Environments (WiSEE).

[5] Aragbaiye Y.M., Isleifson D. (2023). Mass Reduction Techniques for Short Backfire Antennas: Additive Manufacturing and Structural Perforations. Sensors.

[6] Fan K., Hao Z.C., Yuan Q., Luo G.Q., Hong W. (2020). A Wideband High-Gain Planar Integrated Antenna Array for E-Band Backhaul Applications. IEEE Trans. Antennas Propag..

[7] Li Y., Ge L., Wang J., Da S., Cao D., Wang J., Liu Y. (2019). 3-D Printed High-Gain Wideband Waveguide Fed Horn Antenna Arrays for Millimeter-Wave Applications. IEEE Trans. Antennas Propag..

[8] Jin H., Che W., Chin K.S., Shen G., Yang W., Xue Q. (2017). 60-GHz LTCC differential-fed patch antenna array with high gain by using soft-surface structures. IEEE Trans. Antennas Propag..

[9] Zhu J., Chu C.H., Deng L., Zhang C., Yang Y., Li S. (2018). Mm-wave high gain cavity-backed aperture-coupled patch antenna array. IEEE Access.

[10] Duangtang P., Mesawad P., Wongsan R. Gain improvement of conical horn antennas by adding wire medium structure. Proceedings of the 2016 13th International Conference on Electrical Engineering/Electronics, Computer, Telecommunications and Information Technology (ECTI-CON).

[11] Shrestha S., Baba A.A., Abbas S.M., Asadnia M., Hashmi R.M. (2021). A horn antenna covered with a 3D-printed metasurface for gain enhancement. Electronics.

[12] Kampeephat S., Krachodnok P., Wongsan R. Gain improvement for conventional rectangular horn antenna with additional EBG structure. Proceedings of the 2014 11th International Conference on Electrical Engineering/Electronics, Computer, Telecommunications and Information Technology (ECTI-CON).

[13] Kampeephat S., Krachodnok P., Wongsan R. A study of gain enhancement of horn antenna using EBG. Proceedings of the 2012 IEEE Asia-Pacific Conference on Antennas and Propagation.

[14] Kandan M., Kandasamy J.P., Rao P.H. (2018). Gain enhancement of horn antenna using meta surface lens. Adv. Electromagn..

[15] Zhang Z.Y., Lu K., Leung K.W. (2023). Gain Enhancement of Horn Antenna Using a Metal Lens. IEEE Trans. Antennas Propag..

[16] Liu Y., Isleifson D., Shafai L. (2023). Gain Enhancement and Cross-Polarization Suppression of Cavity-Backed Antennas Using a Flared Ground Cavity and Iris. Sensors.

[17] Tianang E.G., Elmansouri M.A., Filipovic D.S. Wide bandwidth cavity-backed dual-polarized vivaldi array antenna. Proceedings of the 2017 IEEE Antennas and Propagation Society International Symposium, Proceedings.

[18] Harrington R.F. (1960). Effect of antenna size on gain, bandwidth, and efficiency. J. Res. Natl. Bur. Stand. Sect. D Radio Propag..

[19] Wong K.L., Tang C.L., Chiou J.Y. (2002). Broad-band probe-fed patch antenna with a W-shaped ground plane. IEEE Trans. Antennas Propag..

[20] Mansoori A., Isleifson D., Shafai L. (2022). Improving Compact Short Backfire Antenna Gain and Cross-Polarization using Choke and Ring Cavity Loading. IEEE Trans. Antennas Propag..

[21] Pathan R., Tripathi A. Inclined Slots Waveguide Antenna Design. 2020. https://ssrn.com/abstract=3573535.

[22] Nessel J.A., Kory C.L., Lambert K.M., Acosta R.J., Miranda F.A. A microstrip patch-fed short backfire antenna for the tracking and data relay satellite system—Continuation (TDRSS-C) Multiple Access (MA) array. Proceedings of the 2006 IEEE Antennas and Propagation Society International Symposium.

[23] Zárate Y.D., Torres F., Rodriguez M., Pizarro F. (2023). 3D-printed low-cost choke corrugated Gaussian profile horn antenna for Ka-band. Sci. Rep..

[24] Qi Z., Li X., Xiao J., Zhu H. (2019). Gain and Bandwidth Improvement of Empty Substrate Integrated Waveguide H-plane Horn Antenna at W-band. J. Infrared Millim. Terahertz Waves.

[25] Binion J.D., Lier E., Hand T.H., Jiang Z.H., Werner D.H. (2019). A metamaterial-enabled design enhancing decades-old short backfire antenna technology for space applications. Nat. Commun..

[26] Wang J., Xu Z., Wang Z., Zheng X., Rahmat-Samii Y. (2023). Development of a Low-Cost Lightweight Advanced K-Band Horn Antenna With Charge-Programmed Deposition 3D Printing. IEEE Antennas Wirel. Propag. Lett..

[27] Ishchenko E.A., Pasternak Y.G., Pendyurin V.A., Rogozin E.A., Fedorov S.M. (2021). Horn antenna with integrated metamaterial for beam steering. J. Phys. Conf. Ser..

[28] Pasternack (2017). WR-284 Waveguide Standard Gain Horn Antenna. https://www.pasternack.com/images/ProductPDF/PE9860-SF-15.pdf.

